# Analysis of incident reports in information systems for patient safety management

**DOI:** 10.1590/0034-7167-2025-0630

**Published:** 2026-10-16

**Authors:** Renata Lima Gonçalves Caroccini, Geisa Colebrusco de Souza Gonçalves, Elena Bohomol

**Affiliations:** I Universidade Federal de São Paulo. São Paulo, São Paulo, Brazil

**Keywords:** Health Information Systems, Risk Management, Patient Safety, Accreditation, Quality of Health Care., Sistemas de Información en Salud, Notificación, Seguridad del Paciente, Acreditación de Instituciones de Salud, Calidad de la Atención de Salud

## Abstract

**Objectives:**

to analyze the characteristics of reports of serious incidents and deaths, as well as the improvement actions implemented, considering organizational learning in patient safety management.

**Methods:**

documentary, descriptive, and quantitative research, conducted in a private electronic system for adverse event reports of an accredited organization. Descriptive and inferential statistics were used for association analysis.

**Results:**

reports were mostly from hospital services (98.8%), from the Southeast region (76.7%), from level 1 accredited institutions (36.7%), made after 30 days of the event (49.4%), and occurred with older patients (48.2%). As an organizational consequence of the incidents, there was additional use of resources, treatment, and examinations (54.3%). Among the improvement actions related to professionals, informal guidance stood out (43.9%), and to organizational factors, actions to improve safety culture (31.9%).

**Conclusions:**

the reported events did not present objective information about institutional learning and implementation of actions for care quality and safety.

## RESUMEN

**Objetivos:**

analizar las características de las notificaciones de incidentes graves y fallecimientos, así como las acciones de mejora implementadas, considerando el aprendizaje organizacional en la gestión de la seguridad del paciente.

**Métodos:**

investigación documental, descriptiva y cuantitativa, realizada en un sistema electrónico privado de notificación de eventos adversos de una organización acreditada. Se utilizaron estadísticas descriptivas e inferenciales para el análisis de asociación.

**Resultados:**

las notificaciones procedían principalmente de servicios hospitalarios (98,8%), de la región Sudeste (76,7%), de instituciones acreditadas de nivel 1 (36,7%), se realizaron después de 30 días del evento (49,4%) y ocurrieron con pacientes de edad avanzada (48,2%). Como consecuencia organizativa de los incidentes, se observó un mayor uso de recursos, tratamientos y exámenes (54,3%). Entre las acciones de mejora relacionadas con los profesionales, destacó la orientación informal (43,9%), y en cuanto a los factores organizativos, las acciones para mejorar la cultura de seguridad (31,9%).

**Conclusiones:**

los eventos reportados no aportaron información objetiva sobre el aprendizaje institucional ni sobre la implementación de acciones para la calidad y seguridad de la atención.

## INTRODUCTION

The healthcare field has implemented numerous actions that have enabled progress in quality improvement and patient safety processes. However, much remains to be done^(1)^. Evidence of this is the World Health Organization (WHO) Global Patient Safety Action Plan 2021-2030, which expands patient safety and proposes an organized framework to minimize risks and preventable harm, making errors less likely and, when they occur, reducing their impact^(2)^. Specifically, strategic objective 6 of the document reinforces the importance of ensuring a continuous flow of information and knowledge to reduce risks and preventable harm and improve the safety of care^(2)^. In its first interim report, WHO highlighted global healthcare strategies, showing that 32% of countries have 60% or more of their institutions with reporting systems, and 38% have adopted reporting of never events or sentinel events^(3)^.

The Brazilian National Health Regulatory Agency established, starting in 2006, the Health Surveillance Reporting System (Notivisa - *Sistema de Notificações de Vigilância Sanitária*), whose purpose was to centralize reports nationwide, initially for technical complaints related to health products and later for serious adverse events and deaths, in accordance with the Brazilian National Patient Safety Program (PNSP - *Programa Nacional de Segurança do Paciente*)^(4,5)^.

Computerized reporting systems support decision-making regarding quality and safety by recording events, supporting analyses, and monitoring actions to reduce recurrence^(6)^. WHO recommends developing systems that allow the inclusion of fields and data such as patient and incident characteristics, consequences, involved agents, contributing and mitigating factors, and implemented improvement actions, constituting an institutional learning process^(7)^.

There are significant particularities regarding how each country, considering the global context, or each institution, considering the national or local context, implements and/or integrates safety indicators into its information systems^(3)^, in data management and access, and how these reflect institutional performance in patient safety policies^(3,7)^. In this direction, accrediting services and institutions have created their own incident reporting systems, such as the National Accreditation Organization (ONA - *Organização Nacional de Acreditação*), which in 2013 created the proprietary system *Integrare*, according to Guideline 21 so that certified healthcare institutions can report incidents or near misses, with or without harm, for monitoring and risk management. Reporting of serious events, with permanent physical or psychological harm or death, must be carried out within 72 hours, either in a proprietary system or in Notivisa^(8)^.

Considering that organizational learning can occur from reported incidents, this study sought to answer the following questions: what are the characteristics of the reports contained in the information system of an accrediting organization, and what improvement actions are implemented by accredited services based on reported incidents?

### Study relevance

This article is the product of a dissertation^(9)^ deposited in the institutional repository. https://repositorio.unifesp.br/home.

## OBJECTIVES

To analyze the characteristics of reports of serious incidents and deaths, as well as the improvement actions implemented, considering organizational learning in patient safety management.

## METHODS

### Ethical aspects

The project received approval from ONA’s Technical and Administrative Board. It was reviewed and approved by the Research Ethics Committee of the proposing institution. The researchers signed a confidentiality agreement regarding the raw system data, committing not to disclose data that could identify services, professionals, and/or patients involved in the analyzed reports. Regarding the analytical tools used in the study, one is publicly available^(10)^, and the other, *Basado en Análisis Causa-Raiz* (BACRA)^(11)^, was authorized by the authors.

### Study design, period, and setting

This is a documentary, descriptive study with a quantitative approach, conducted based on incidents reported in the proprietary electronic system *Integrare* ONA. The STrengthening the Reporting of OBservational studies in Epidemiology was used to guide the study.

*Integrare* stores and organizes reports as a primary database. Access to all fields is restricted, with confidential interfaces limited to ONA. After reporting, a team conducts analysis together with accredited services and institutions to promote organizational learning from reported incidents. The system includes free-text fields without limits or mandatory sequential completion, allowing repetition of information across fields and resulting in an unstructured textual format. Additional textual fields are used by ONA analysts to monitor post-event improvement actions in healthcare services. The analyzed information includes incidents reported by professionals and follow-up actions conducted by the accrediting body between 2013 and 2018. These data were provided to the researchers in August 2019 in a Microsoft Excel^®^ spreadsheet. The researchers did not interact with the system or access restricted organizational data.

### Population or sample; inclusion and exclusion criteria

The analyzed database contained 1,307 reports, which were fully reviewed. A subset of reports with serious outcomes and deaths was selected, totaling 509 reports. Five duplicate reports were excluded, and 65 were improperly classified, resulting in a final sample of 439 reports, of which 151 (34.4%) were serious and 288 (65.6%) were deaths.

### Study protocol

Given the reporting format and study objectives, two frameworks were used to categorize the reports into variables: the Conceptual Framework for the International Classification for Patient Safety (ICPS)^(10)^ and BACRA^(11)^.

Classification and categorization were performed in a Microsoft Excel^®^ spreadsheet using the following variables: 1. Type of healthcare service; 2. Geographic location; 3. Institution certification level; 4. Time interval from incident to reporting; 5. Patient’s life stage; 6. Intrinsic patient risk factors (frailty, comorbidities, extremes of age); 7. Type of cause (immediate - when active errors are made by professionals in direct contact with patients; or latent/system failure - less evident circumstances and failures within organizational processes and system design); 8. Nature of harm (related to the type of procedure); 9. Detection of occurrence; 10. Organizational consequences (post-event actions related to care delivery); 11. Improvement actions related to professionals (post-event actions involving professionals); and 12. Actions to reduce the risk of organizational factors (implemented actions and barriers at the organizational level). When not identified, it was classified as absent.

The reading and categorization were performed by one researcher and subsequently verified independently by two others, with disagreements resolved by consensus.

### Analysis of results and statistics

Descriptive analysis was performed, including absolute and relative frequencies for all variables. Associations were investigated between certification level and type of cause with nature of harm, improvement actions related to professionals, and actions to reduce organizational risk. Pearson’s chi-square test was used to assess associations and compare proportions. Post-hoc analysis was conducted using the z-test. Statistical significance was set at p ≤ 0.05. Analyses were performed using the Statistical Package for the Social Sciences version 26.0.

## RESULTS

Of the 439 reports included in the final sample, incident outcomes were death in 288 (65.6%) cases and serious harm in 151 (34.4%). Thus, 434 (98.8%) were from hospital services, 335 (76.7%) were from the Southeast region, 161 (36.7%) were from institutions with certification level 1, and 109 (49.4%) occurred more than 30 days after the incident, as shown in Table 1.

**Table 1:** Distribution of reported incidents by service type, Brazilian region, certification level, and time interval, São Paulo, São Paulo, Brazil, 2023

Variables	N total (439)	%
Type of service		
Hospital	434	98.8
Diagnostic imaging	2	0.5
Outpatient clinic	2	0.5
Renal replacement therapy	1	0.2
Brazilian region		
Southeast	335	76.4
Central-West	48	11
South	30	6.8
Northeast	16	3.6
North	8	1.8
No region information	2	0.4
Certification level		
Level 1 - Accredited	161	36.7
Level 2 - Fully accredited	149	33.9
Level 3 - Accredited with excellence	129	29.4
Reporting time interval		
> 30 days	217	49.4
11-30 days	109	24.8
4-10 days	83	18.9
0-3 days	30	6.9

Reports occurred predominantly with older patients (160; 48.2%). Frailty was the main intrinsic factor attributed to 269 (67.1%) patients. Concerning the type of cause, 301 (70.3%) were categorized as immediate, when there was a direct relationship between the professional and the patient. As for the nature of harm, delay in detecting clinical deterioration and delay in surgical intervention were found in 64 (15.5%) patients. The occurrences were detected by 341 (94.2%) healthcare professionals (Table 2).

**Table 2:** Distribution of reported incidents by life stage, intrinsic patient factors, type of incident cause, and nature of harm, São Paulo, São Paulo, Brazil, 2023

Variables	n	%
Life stage (n=332)		
Older adult (over 60 years)	160	48.2
Adult (18-60 years)	107	32.2
Child (0-12 years)	50	15
Pregnant ^†^	13	4
Adolescent (12-18 years)	2	0.6
Intrinsic patient risk factors (n=401)		
Individuals’ frailty	269	67.1
Comorbidities	76	19
Extremes of age (over 85 years and premature infants)	17	4.2
Impaired mobility and physical dependence	15	3.7
Poor communication with professionals	11	2.8
Uncooperative attitude (agitation/delirium)	9	2.2
Clinical deterioration	4	1
Causes (n=428)		
Immediate	301	70.3
Latent	127	29.7
Nature of harm (n= 413)		
Delay in detecting clinical deterioration	64	15.5
Delay in surgical intervention	64	15.5
Medication-related	59	14.3
Related to the treatment of respiratory failure	56	13.5
Healthcare-associated infections	38	9.2
Fall	17	4.1
Probing procedure failures	14	3.4
Delay in test results	13	3.2
Pneumothorax	11	2.7
Hemorrhage	10	2.4
Other	67	16.2
Detection of occurrence (n=362)		
Professionals involved in care	341	94.2
Family members	12	3.3
People involved in spiritual assistance	5	1.4
Institutional committees	4	1.1

† Optou-se por destacar o termo “gestante”, visto ter sido utilizado nas notificações como marcador do grupo ao qual pertencia a notificação ao invés de classificar por idade.

Table 3 presents the distribution of incidents classified according to post-event organizational consequences. There was an additional increase in resources for treatment/examinations related to the incident in 190 (54.3%) reports, in addition to admission to the critical care area, with an increase in length of stay in the institution in 152 (43.4%). Concerning improvement actions related to professionals, informal guidance was identified in 133 (43.9%) adverse event descriptions. In relation to actions triggered to reduce the risk of organizational factors, it was found that 87 (31.9%) of descriptions generated actions to improve safety culture related to error and hazardous condition reporting, case review, debriefing, and complaint management.

**Table 3:** Distribution of reported incidents by organizational consequences, professional-related improvement actions, and actions to reduce the risk of organizational factors, São Paulo, São Paulo, Brazil, 2023

Variables	n	%
Organizational consequences (n=350)		
Additional resources for treatment and examinations	190	54.3
Admission to the critical care unit and increased length of stay	152	43.4
Legal implications	7	2
Formal complaint	1	0.3
Improvement actions related to professionals (n=303)		
Informal guidance	133	43.9
Availability of protocol and guideline checklist	70	23.1
Education and training	53	17.5
Supervision	44	14.5
Staffing levels adequate	3	1
Actions to reduce the risk of organizational factors (n=273)		
Actions to improve safety culture	87	31.9
Staff suitability and qualification for tasks	68	24.9
Quick access to decision support protocols	58	21.2
Leadership commitment	30	11
Compliance with codes and regulations	19	7
Implementation of risk assessments	11	4

In the associative analyses, Table 4 shows a statistically significant association between the type of cause (immediate and latent) and the type of improvement action related to professionals. Post-hoc analysis revealed a difference only in “education and training”, meaning a higher occurrence of “education and training” type improvements in immediate causes compared to latent causes. No statistically significant associations were found between the type of cause and the nature of damage, nor between the type of cause and actions to reduce risk. Organizational factors, latent causes, and immediate causes were similar in relation to these variables.

**Table 4:** Analysis of the association between the type of cause (immediate and latent) and the type of improvement actions related to professionals, São Paulo, São Paulo, Brazil, 2023

Variables	Immediate cause	Latent cause	Total	*p* value
	n/%	n/%	n/%	
Types of improvement actions				
Guidelines	89/29.6	42/33.1	131/30.6	0.002^†^
Availability of protocol and guideline checklists	46/15.3	22/17.3	68/15.9	
Education and training	50/16.6	3/2.4	53/12.4	
Supervision	29/9.6	14/11	43/10	
Adequacy of staffing levels	3/1	0/0	3/0.7	

† Pearson’s chi-square test; statistically significant value at the 5% level (p ≤ 0.05).

In the other associative analyses, there was no statistically significant association between the level of accreditation, the nature of damage, the type of improvement action related to professionals, and the type of action to reduce organizational risk.

## DISCUSSION

In this study, characteristics of events and aspects related to implemented improvement actions, additional resource use after the event, and the fact that most improvement actions related to professionals consisted of informal guidance were identified, while actions related to the organization were associated with safety culture. It was also found that narrative reports, although advantageous for detailing events, make analysis and understanding of outcomes for organizational learning more difficult.

Reporting systems are important tools for promoting organizational learning. Despite their various benefits, managers face difficulties in analyzing and monitoring reported events, especially when institutions adopt a punitive approach without implementing necessary changes to improve care or prioritizing organizational learning from incidents^(6)^.

The need for efficient reporting systems has been widely highlighted as a key action in transforming safety culture. Their ineffectiveness results in heterogeneous interventions and measures in the pursuit of safety and quality in healthcare services^(12)^.

Regarding the characteristics of the analyzed events, it was expected that most would occur in hospital services, environments characterized by acute conditions and high technological complexity, especially since most accredited services in Brazil are located in the Southeast region, as reported in the literature^(13,14)^. These data were confirmed by a study of Notivisa reports, which showed a higher number of records in healthcare services in the Southeast region^(15)^.

It was observed that half of the reports occurred more than 30 days after the event, indicating that they were entered into the system only after analysis had been completed. For comparison, Guideline 21^(8)^ establishes a five-day reporting deadline from the event date for severe harm or death, which was the focus of this study. Additionally, in Brazil, regardless of certification or internal systems, the PNSP recommends that adverse events resulting in death be reported within 72 hours, followed by the inclusion of analytical information^(5)^.

Regarding patient characteristics, a higher occurrence was identified among older patients, with frailty defined as the main intrinsic factor, resulting from the accumulation of multiple functional deficits. This finding is not coincidental, as population aging increases care challenges and leads to a higher proportion of older patients in healthcare services. Adverse events have also been identified as more frequent among hospitalized older patients, with greater risk of severity or mortality^(16)^.

Although “frailty” is a complex and emerging concept, it is often associated with older adults. Several instruments can facilitate its identification in patients undergoing medical-surgical care and support healthcare professionals in clinical decision-making and the adoption of specific protocols, functioning as a barrier to prevent adverse events and severe harm^(17)^.

The classification of immediate causes predominated in this study, involving frontline professionals directly engaged in patient care, such as nursing, medical, and physiotherapy staff. In this context, safety culture may reflect values and attitudes that determine how professionals perform their work and relate to quality improvement processes, incorporating preventive strategies and interventions to reduce events^(18)^. Regarding latent causes, researchers highlight working conditions, such as structure and processes, that expose healthcare professionals to potential, generally preventable risks, thus requiring attention from leadership for the implementation of active and effective risk management^(19)^.

In this regard, there is a need to include barrier resources and triggers related to patient profiles and to identify the nature of harm associated with commonly observed adverse events. The findings highlight that failure to identify clinical deterioration and delays in surgical intervention resulted in unexpected patient death. A systematic review with meta-analysis investigated the relationship between adverse events, patient characteristics, and hospital characteristics, identifying an incidence of 8.6 events per 100 hospitalizations, of which 50% were preventable. Among older adults, events associated with surgical procedures, medication use, and healthcare-associated infections were highlighted^(20)^, aspects also analyzed in this study.

For organizational learning, recognition by professionals of what constitutes an adverse event and the confidence to report it without fear of punishment are fundamental conditions, as most reports were made by healthcare professionals. Currently, there is greater awareness of incidents and adverse events, with leadership focused on quality and patient safety. Clarifying causes and implementing barriers to prevent recurrence, with the involvement of the care team, is essential^(21)^.

Incident management results in negative consequences for all involved and for the organization, as well as increased costs, use of structural and material resources, and longer patient stays. A study conducted in Japan reinforces that patient safety reports aim to develop better policies that promote prosperity, equity, opportunity, and well-being. The same study highlighted the importance of investigating the cost-effectiveness of safety measures, as 15% of hospital expenditures are attributed to the treatment of care-related failures, such as intraoperative injuries, incorrect medication administration, and falls^(1)^. Thus, patient safety should be addressed not only as a public health issue but also from an economic and financial sustainability perspective.

Another fundamental aspect is the role of patients and families as agents of their own care, particularly in recognizing risks in care delivery. When a patientand family-centered care model is adopted, their participation in ensuring care quality is acknowledged^(22,23)^.

As a central finding of this study, it is noteworthy that improvement actions related to professionals and to reducing organizational risk factors are incipient, as most event descriptions and analyses did not specify which actions were implemented after the incident. Although the accreditation process includes elements that promote safety culture, such as event recording and analysis, mitigation actions should be more robust and monitored after the event^(15,19)^.

It is also important to highlight the broad use of the safety culture concept based on ICPS, which makes it difficult to clearly define what is being measured and whether safety culture is present or absent. Although institutions and their professionals were included in the analysis, they took responsibility for identifying, reporting, and resolving safety issues such as adverse events^(10)^. However, there remains a gap, particularly regarding risk resolution, which should involve improvement actions to prevent recurrence.

There was also an association between education and training in occurrences of immediate causes compared to latent causes. It is emphasized that professionals learn from their own experiences. Educational activities and the implementation of specific protocols are improvement actions that function as prevention and effective barriers to patient safety^(23)^. Continuous educational actions should be reinforced as a key pillar in preventing adverse events, as direct involvement of healthcare professionals and ongoing training enhance safety for both patients and professionals. Despite difficulties in reporting due to system limitations or organizational culture, professionals recognize that reporting supports continuing education^(5)^.

It is worth noting that, although a robust training program is offered to professionals, individual perception and knowledge retention vary, as each person brings with them different perspectives and ingrained practices, according to their professional category. For them to work harmoniously, respecting the limits of their responsibilities, effective supervision and leadership within the team is essential^(24)^, ensuring adequate conditions for care.

When processing the analysis of reports constructed from open text fields, it was found that the content of the reports mentioned, in addition to the event itself, situations related to the organizational dynamics, such as insufficient service structure, inadequate working conditions, absence of verification stages related to patient care, errors in clinical documentation, weaknesses related to teamwork, failures related to communication with the patient or the information system, and lack of risk assessment, among other situations that could constitute barriers to the prevention of the event. Although such causes may be considered generic, they contribute to the occurrence of incidents because they are part of the institution’s daily routine, jeopardizing patient care and involving frontline professionals^(25)^.

Given the missing data in the analyses for the composition of the variables in this study, the importance of having a computerized system with structured reports, clear guidelines on what and how to report, what actions were implemented and their monitoring, with specific indicators for organizational development, whose data can generate meaningful learning, is reinforced. It is also necessary that the system be easy to use and provide feedback to the community in general^(3,7)^. Furthermore, delays in providing feedback after analyzing the reporting can delay preventive measures^(7)^.

Therefore, reporting system should be built to facilitate reporting by the person making the report and directed towards later analysis, if organizational learning is desired^(6,26)^. A hybrid structure is suggested, with drop-down lists to give objectivity to the report, and open fields so that they can be used to supplement the information. In this research, it was identified that reporting in a system with only open fields may neglect important information that is not recorded. Strategies to increase reports and their understanding are found in the literature. The hospital managed to encourage the reporting of adverse events and establish a new communication channel to improve the organizational environment through QR Code^(27)^.

Despite the importance of system-based reports, the study highlights the importance of obtaining information on adverse events through other methodologies as well, such as the Global Trigger Tool or retrospective medical record analysis^(28)^.

### Study limitations

This study in question achieved its objective of analyzing reports of incidents classified as serious and deaths in the patient safety information system, as well as understanding institutional learning. Among its limitations are the analysis of events reported narratively, in an open field setting, which allows for numerous deviations and distances from the objective fact, as well as the incorporation of value judgments by the professionals who make the reports, not always elucidating what occurred and the actions taken. Furthermore, despite the concern to standardize the classification and resolve divergences among researchers, it is impossible to eliminate all biases due to the nature of the analysis, which is anchored in personal values and prior experiences.

### Contributions to the Field

By analyzing adverse event reports that resulted in serious injury or death, it is possible to identify critical points and vulnerabilities in care, administrative, or management processes. However, this identification does not always translate into learning opportunities and improvement actions after the event, which strengthens the safety culture.

## CONCLUSIONS

The incident reports analyzed, with serious outcomes and deaths, from the accrediting organization’s own information system, showed that, although there is no single model for these systems, the absence of criteria for documenting the incident impacts post-event analysis and makes it difficult to understand how organizational learning occurred.

In the event of adverse events, there is additional use of resources. Improvement actions were absent in most analyses, identifying weaknesses in learning processes and organizational maturity. In general, improvement actions related to professionals were characterized as informal guidance, and improvement actions related to the organization refer to the safety culture, although this may mean a diversity of actions.

The classification of the cause as immediate was predominant, and it involved frontline professionals in direct patient care, with no description of support actions for professionals regarding the outcome and the resulting suffering.

While there are advantages to a reporting system allowing for narrative reporting, explaining in detail what occurred, this format presents the disadvantage of difficulty in analyzing the information to identify necessary actions to prevent recurrence. Therefore, it is crucial that the reporting system be structured to facilitate both reporting and subsequent analysis of information, promoting continuous process improvement and organizational learning.

## Data Availability

The research data are not available.
