# Supplementary material for: Analysis of incident reports in information systems for patient safety management
Source: Rev Bras Enferm. 2026 Oct 16;79:e20250630. doi: 10.1590/0034-7167-2025-0630 (PMC13679171; doi:10.1590/0034-7167-2025-0630)
Supplement: Translation

# Translation: Análise de notificação dos incidentes em sistemas de informação para gestão da segurança do paciente

**DOI:** 10.1590/0034-7167-2025-0630pt

**Authors:** Renata Lima Gonçalves Caroccini, Geisa Colebrusco de Souza Gonçalves, Elena Bohomol

**Affiliations:** I Universidade Federal de São Paulo. São Paulo, São Paulo, Brasil

**Keywords:** Sistemas de Informação em Saúde, Notificação, Segurança do Paciente, Acreditação de Instituições de Saúde, Qualidade da Assistência à, Saúde.

## Abstract

**Objetivos:**

analisar as características das notificações de incidentes graves e óbitos, bem como as ações de melhoria implementadas, considerando a aprendizagem organizacional na gestão da segurança do paciente.

**Métodos:**

pesquisa documental, descritiva e quantitativa, realizada em sistema eletrônico privativo de notificações de eventos adversos de organização acreditadora. Utilizaram-se estatística descritiva e inferencial para análise de associações.

**Resultados:**

as notificações eram majoritariamente de serviços hospitalares (98,8%), da região Sudeste (76,7%), de instituições acreditadas no nível 1 (36,7%), realizadas após 30 dias do evento (49,4%), ocorridas com pacientes idosos (48,2%). Como consequência organizacional dos incidentes, houve uso adicional de recursos, tratamento e exames (54,3%). Entre as ações de melhoria relacionadas aos profissionais, destacaram-se orientações informais (43,9%), e aos fatores organizacionais, ações de melhoria da cultura de segurança (31,9%).

**Conclusões:**

os eventos notificados não apresentaram informações objetivas acerca da aprendizagem institucional e da implementação de ações para a qualidade e segurança do cuidado.

## INTRODUÇÃO

A área da saúde instituiu inúmeras ações que permitiram avançar nos processos de melhoria da qualidade e do segurança do paciente. No entanto, ainda há muito o que fazer^(1)^. Prova disso é o Plano de Ação Global 2021-2030 da Organização Mundial da Saúde (OMS), que amplia a segurança do paciente e propõe uma estrutura organizada para minimizar riscos e danos evitáveis, tornando os erros menos prováveis e, quando ocorrem, que tenham menor impacto^(2)^. Especificamente, o objetivo estratégico seis do documento reforça a importância de se garantir fluxo contínuo de informações e conhecimentos para reduzir riscos e danos evitáveis e melhorar a segurança dos cuidados^(2)^. No primeiro relatório parcial, a OMS destacou estratégias globais de cuidados de saúde, mostrou que 32% dos países têm 60% ou mais de seus estabelecimentos com sistemas de notificação e 38% adotaram notificações de *never events* ou eventos sentinela^(3)^.

No Brasil, a Agência Nacional de Vigilância Sanitária criou, a partir de 2006, o Sistema de Notificações de Vigilância Sanitária (Notivisa), cuja finalidade foi estabelecer, em território nacional, a centralização de notificações, a princípio para queixas técnicas de produtos de saúde e, posteriormente, para eventos adversos graves e óbitos, conforme o Programa Nacional de Segurança do Paciente (PNSP)^(4,5)^.

Sistemas informatizados de notificação auxiliam na decisão sobre qualidade e segurança, registram eventos, apoia análises e monitoram ações para reduzir recorrências^(6)^. A OMS orienta que sejam construídos sistemas que permitam a inclusão de campos e dados, como características do paciente e incidente, consequências, agentes envolvidos, fatores contribuintes e mitigantes, e ações de melhoria implementadas, constituindo um processo de aprendizagem institucional^(7)^.

Há particularidades significativas sobre como cada país, no contexto global, ou cada instituição, considerando o contexto nacional e local, implementa e/ou integra indicadores de segurança nos seus sistemas de informação^(3)^, no gerenciamento e no acesso a dados, e sobre como eles refletem o desempenho institucional nas políticas de segurança do paciente^(3,7)^. Nesta direção, serviços e instituições acreditadoras têm criado o próprio sistema de notificação de incidentes, a exemplo da Organização Nacional de Acreditação (ONA), que criou, em 2013 o sistema privativo *Integrare,* conforme Norma Orientadora nº 21, para que instituições de saúde por ela certificadas realizem notificações de incidentes ou quase incidentes, com ou sem danos, para acompanhamento e gestão de risco. A notificação de eventos graves, com danos físicos, psicológicos permanentes ou óbitos, deve ser feita em até 72 horas, em sistema próprio ou no Notivisa^(8)^.

Considerando que a aprendizagem organizacional pode ocorrer a partir dos incidentes notificados, pretendeu-se responder às perguntas: quais as características das notificações contidas no sistema de informação de uma organização acreditadora e quais são as ações de melhoria implementadas pelos serviços acreditados a partir dos incidentes notificados?

### Relevância do estudo

Este artigo é produto de dissertação^(9)^ depositada no repositório institucional https://repositorio.unifesp.br/home.

## OBJETIVOS

Analisar as características das notificações de incidentes graves e óbitos, bem como as ações de melhoria implementadas, considerando a aprendizagem organizacional na gestão da segurança do paciente.

## MÉTODOS

### Aspectos éticos

O projeto recebeu anuência e aprovação da Superintendência Técnica e Administrativa da ONA. Foi analisado e aprovado pelo Comitê de Ética em Pesquisa da instituição proponente. As pesquisadoras assinaram um Termo de Confidencialidade e Sigilo das informações dos dados brutos do sistema, comprometendo-se a não divulgar dados que possam identificar serviços, profissionais e/ou pacientes envolvidos nas notificações analisadas. Acerca das ferramentas de análise utilizadas no estudo, uma é de acesso livre^(10)^, e a outra, *Basado en Análisis Causa-Raiz* (BACRA)^(11)^, foi autorizada pelos autores responsáveis.

### Desenho, período e local do estudo

Pesquisa documental, descritiva, de abordagem quantitativa, realizada a partir dos incidentes notificados no sistema eletrônico privativo *Integrare* ONA. Utilizou-se o *STrengthening the Reporting of OBservational studies in Epidemiology* para guiar a pesquisa.

O *Integrare* armazena e organiza as notificações como um banco primário de dados. Tem acesso limitado à totalidade dos campos, com interfaces confidenciais à própria ONA. Após a notificação, uma equipe realiza a análise em conjunto com serviços e instituições acreditadoras credenciadas para promover aprendizado organizacional em relação aos incidentes notificados. Contém campos de informações em texto livre, sem limite ou obrigatoriedade de preenchimento sequencial pelos profissionais de saúde, com possibilidade de repetir informações em diversos campos, construído de forma textual e não estruturado. Outros campos, também em formato textual, complementam o sistema de preenchimento dos analistas da ONA com o objetivo de monitorar ações de melhoria dos serviços de saúde pós-evento. As informações analisadas incluem incidentes relatados pelo profissional e o acompanhamento realizado pela certificadora após o evento, entre os anos de 2013 e 2018. Essas informações foram cedidas em agosto de 2019 aos pesquisadores em uma planilha do *Microsoft Excel* ^®^, em um dispositivo de armazenamento de dados. Os pesquisadores não interagiram com o sistema nem visualizaram informações restritas da organização acreditadora.

### População ou amostra; critérios de inclusão e exclusão

O banco de dados analisado nesta pesquisa totalizava 1.307 notificações, que foi lido na íntegra, com recorte de notificações com desfechos graves e óbitos, totalizando 509 notificações. Foram excluídas cinco notificações duplicadas e 65 foram previamente classificadas indevidamente, perfazendo uma amostra final de 439 notificações, sendo que, dessas, 151 (34,4%) eram graves e 288 (65,6%) eram óbitos.

### Protocolo do estudo

Diante do formato da notificação e dos objetivos da pesquisa, escolheram-se dois referenciais para categorizar as notificações em variáveis: Estrutura Conceitual para Classificação Internacional de Segurança do Paciente (CISP)^(10)^ e a ferramenta BACRA^(11)^.

A classificação e a categorização das notificações foram procedidas em planilha do *Microsoft Excel* ^®^ com as seguintes variáveis: 1. Tipo de serviço de saúde; 2. Localização geográfica; 3. Nível de certificação da instituição; 4. Intervalo de tempo do incidente até a notificação; 5. Fase da vida do paciente; 6. Fatores de risco intrínsecos do paciente (fragilidade, comorbidades, extremos de idade); 7. Tipo de causa (imediata - quando os erros ativos são cometidos por profissionais em relação direta aos pacientes; ou latente/falhas do sistema - circunstâncias e falhas menos claras presentes na organização e no *design* de dispositivos e de atividades); 8. Natureza do dano (relacionado ao tipo de procedimento); 9. Detecção da ocorrência; 10. Consequências organizacionais (ações pós-evento decorrentes da prestação de cuidados); 11. Ações de melhoria relacionadas aos profissionais (ações pós-evento com profissionais); e 12. Ações para reduzir o risco dos fatores organizacionais (ações e barreiras implementadas relacionadas à organização). Quando não identificada, classificou-se como informação ausente.

A leitura e a categorização das notificações foram feitas por uma pesquisadora e, em seguida, verificadas por outras duas de forma independente, sendo as divergências resolvidas em reuniões de consenso.

### Análise dos resultados e estatística

Procedeu-se à análise descritiva, frequência absoluta e relativa de todas as variáveis. Foram investigadas associações entre o nível de certificação e o tipo de causa com a natureza do dano, ações de melhoria relacionada aos profissionais e ações para reduzir o risco dos fatores organizacionais. Para verificar a associação e comparar as proporções entre as variáveis, tipo de causa com natureza do dano, ações de melhoria e ações para reduzir o risco, utilizou-se o teste qui-quadrado de Pearson. A análise *post-hoc* foi conduzida com o teste z. Adotou-se o valor de significância estatística p ≤ 0,05. As análises foram realizadas no *software Statistical Package for the Social Sciences*, versão 26.0.

## RESULTADOS

Das 439 notificações que compuseram a amostra final analisada, os desfechos do incidente foram morte, em 288 (65,6%) notificações, e danos graves, em 151 (34,4%). Assim, 434 (98,8%) eram de serviços hospitalares, 335 (76,7%) eram da região Sudeste, 161 (36,7%) eram de instituições com nível 1 de certificação, e 109 (49,4%) ocorreram após 30 dias da ocorrência, conforme observado na Tabela 1.

**Tabela 1:** Distribuição dos incidentes notificados por tipo de serviço, região brasileira, nível de certificação e intervalo de tempo, São Paulo, São Paulo, Brasil, 2023

Variáveis	N total (439)	%
Tipo de serviço		
Hospitalar	434	98,8
Diagnóstico por imagem	2	0,5
Ambulatório	2	0,5
Terapia renal substitutiva	1	0,2
Região brasileira		
Sudeste	335	76,4
Centro-Oeste	48	11
Sul	30	6,8
Nordeste	16	3,6
Norte	8	1,8
Sem informação da região	2	0,4
Nível de certificação		
Nível 1 - Acreditada	161	36,7
Nível 2 - Acreditada plena	149	33,9
Nível 3 - Acreditada com excelência	129	29,4
Intervalo de tempo da notificação		
> 30 dias	217	49,4
11-30 dias	109	24,8
04-10 dias	83	18,9
0-03 dias	30	6,9

As notificações ocorreram majoritariamente com pacientes idosos (160; 48,2%). A fragilidade foi o principal fator intrínseco atribuído a 269 (67,1%) pacientes. Quanto ao tipo de causa, 301 (70,3%) foram categorizadas como imediatas, quando ocorreu relação direta entre profissional e paciente. Quanto à natureza do dano, o atraso na detecção da deterioração clínica e o atraso da intervenção cirúrgica foram constatados em 64 (15,5%) pacientes. As ocorrências foram detectadas por 341 (94,2%) profissionais assistenciais (Tabela 2).

**Tabela 2:** Distribuição dos incidentes notificados por fase da vida, fatores intrínsecos ao paciente, tipo de causa dos incidentes, natureza do dano, São Paulo, São Paulo, Brasil, 2023

Variáveis	n	%
Fase da vida (n=332)		
Idoso (acima de 60 anos)	160	48,2
Adulto (18-60 anos)	107	32,2
Infância (0-12 anos)	50	15
Gestante ^†^	13	4
Adolescência (12-18 anos)	2	0,6
Fatores de risco intrínsecos ao paciente (n=401)		
Fragilidade do indivíduo	269	67,1
Comorbidades	76	19
Extremos da idade (acima de 85 anos e prematuros)	17	4,2
Mobilidade prejudicada e dependência física	15	3,7
Má comunicação com profissionais	11	2,8
Atitude não cooperativa (agitação/delírio)	9	2,2
Deterioração clínica	4	1
Causas (n=428)		
Imediatas	301	70,3
Latentes	127	29,7
Natureza do dano (n= 413)		
Atraso na detecção da deterioração clínica	64	15,5
Atraso na intervenção cirúrgica	64	15,5
Relacionados à medicação	59	14,3
Relacionados ao tratamento da insuficiência respiratória	56	13,5
Infecções relacionada à assistência à saúde	38	9,2
Queda	17	4,1
Falhas no procedimento de sondagem	14	3,4
Atraso no resultado de exame	13	3,2
Pneumotórax	11	2,7
Hemorragia	10	2,4
Outros	67	16,2
Detecção da ocorrência (n=362)		
Profissionais envolvidos no cuidado	341	94,2
Familiares	12	3,3
Pessoas envolvidas na assistência espiritual	5	1,4
Comissões institucionais	4	1,1

† Optou-se por destacar o termo “gestante”, visto ter sido utilizado nas notificações como marcador do grupo ao qual pertencia a notificação ao invés de classificar por idade.

Na Tabela 3, apresenta-se a distribuição dos incidentes classificados conforme as consequências organizacionais pós-evento. Houve aumento adicional dos recursos para tratamento/exames relacionados ao incidente em 190 (54,3%) notificações, além de admissão na área de cuidados críticos, com aumento da permanência na instituição em 152 (43,4%). Quanto às ações de melhoria relacionadas aos profissionais, em 133 (43,9%) descrições de eventos adversos, foram identificadas orientações informais. Quanto às ações desencadeadas para reduzir o risco dos fatores organizacionais, constatou-se que 87 (31,9%) das descrições geraram ações para melhoria da cultura de segurança relacionadas à comunicação de erros e condições perigosas, revisão de casos, “*debriefing*” e gestão de reclamações.

**Tabela 3:** Distribuição dos incidentes notificados por consequências organizacionais, ações de melhoria relacionadas aos profissionais e ações para reduzir o risco dos fatores organizacionais, São Paulo, São Paulo, Brasil, 2023

Variáveis	n	%
Consequências organizacionais (n=350)		
Aumento adicional dos recursos para tratamento e exames	190	54,3
Admissão na área de cuidados críticos e aumento do tempo de permanência	152	43,4
Implicações legais	7	2
Reclamação formal	1	0,3
Ações de melhoria relacionadas aos profissionais (n=303)		
Orientações informais	133	43,9
Disponibilidade de checklist de protocolos e diretrizes	70	23,1
Formação e treinamento	53	17,5
Supervisão	44	14.5
Adequação no quantitativo de pessoas	3	1
Ações para reduzir o risco dos fatores organizacionais (n=273)		
Ações para melhoria da cultura de segurança	87	31,9
Adequação e qualificação do pessoal às tarefas	68	24,9
Acesso rápido a protocolos de apoio à decisão	58	21,2
Comprometimento das lideranças	30	11
Cumprimento de códigos e regulamentos	19	7
Implantação de avaliações de risco	11	4

Nas análises associativas, a Tabela 4 mostra associação estatisticamente significativa entre o tipo de causa (imediata e latente) e o tipo de ação de melhoria relacionada aos profissionais, cuja análise *post-hoc* evidenciou diferença apenas em “formação e treinamentos”, ou seja, maior ocorrência de melhorias do tipo “formação e treinamentos” nas causas imediatas, em comparação às causas latentes. Não foram encontradas associações estatisticamente significativas entre o tipo de causa e a natureza do dano, nem entre o tipo de causa e as ações para reduzir o risco. Fatores organizacionais, causas latentes ou imediatas foram semelhantes em relação a essas variáveis.

**Tabela 4:** Análise de associação entre tipo de causa (imediata e latente) e tipo de ações de melhoria relacionadas aos profissionais, São Paulo, São Paulo, Brasil, 2023

Variáveis	Causa imediata	Causa latente	Total	Valor de *p*
	n/%	n/%	n/%	
Tipos de ações de melhoria				
Orientações	89/29,6	42/33,1	131/30,6	0,002^†^
Disponibilidade de checklists de protocolos e diretrizes	46/15,3	22/17,3	68/15,9	
Formação e treinamentos	50/16,6	3/2,4	53/12,4	
Supervisão	29/9,6	14/11	43/10	
Adequação no quantitativo de pessoas	3/1	0/0	3/0,7	

† Teste qui-quadrado de Pearson; valor estatisticamente significante no nível de 5% (p ≤ 0,05).

Nas demais análises associativas, não houve associação estatisticamente significativa entre o nível de acreditação, a natureza do dano, o tipo de ação de melhoria relacionada aos profissionais e o tipo de ação para reduzir o risco organizacional.

## DISCUSSÃO

Nesta pesquisa, identificaram-se características dos eventos e aspectos relacionados às ações de melhoria implementadas, ao uso adicional dos recursos após o evento e ao fato de que a maioria das ações de melhoria relacionadas aos profissionais consistiu em orientações informais, enquanto as ações relacionadas à organização estiveram relacionadas à cultura de segurança. Constatou-se também que notificações narrativas, embora apresente vantagens no detalhamento do evento, dificultam a sua análise e a compreensão dos desdobramentos para a aprendizagem organizacional.

Sistemas de notificação são ferramentas importantes para promover a aprendizagem organizacional. Apesar dos diversos benefícios, destaca-se a dificuldade dos gestores em analisar e monitorar os eventos notificados, especialmente quando as instituições adotam uma abordagem punitiva, sem implementar mudanças necessárias para melhorar a assistência ou priorizar o aprendizado organizacional a partir dos incidentes^(6)^.

A necessidade de sistemas de notificações eficientes tem sido amplamente apontada como ação nuclear na mudança da cultura de segurança. A sua inoperabilidade repercute em intervenções e medidas heterogêneas na busca por segurança e qualidade nos cuidados ofertados em saúde^(12)^.

Quanto às características dos eventos analisados, era esperado, conforme encontrado, que ocorressem majoritariamente em serviços hospitalares e localizados na região Sudeste, local de condições agudas e alta complexidade tecnológica, bem como por serem a maioria dos serviços acreditados no Brasil desta região, conforme aponta a literatura^(13,14)^. Dados semelhantes foram encontrados em estudo de notificações do Notivisa, que apontou maior quantidade de registros em serviços de saúde da região Sudeste^(15)^.

Constatou-se que metade das notificações ocorreu após 30 dias do evento, indicando que foram registradas no sistema somente após a conclusão da análise. Para comparação, a Norma Orientadora nº 21^(8)^ indica como prazo de notificação cinco dias a contar a data do evento para dano grave ou óbito, recorte desta pesquisa. Ainda, no caso brasileiro, independentemente da adoção de certificações ou sistemas internos, o PNSP orienta que eventos adversos que resultarem em óbito devem ser notificados em até 72 horas, com posterior inclusão das informações da análise realizada^(5)^.

No tocante às características dos pacientes envolvidos, identificou-se maior ocorrência com pacientes idosos e a fragilidade como fator intrínseco principal atribuído ao paciente, definida pelo acúmulo de múltiplos déficits funcionais. Característica que não é mera coincidência, pois à medida que a população envelhece mudanças significativas na fisiologia aumentam os desafios na prestação de cuidados e os pacientes idosos passam a ser maior proporção nos serviços. Eventos adversos foram também identificados mais frequentes em pacientes idosos internados em estudo, apresentando maior risco de gravidade ou mortalidade^(16)^.

Embora “fragilidade” seja um conceito complexo e emergente, frequentemente está relacionada às pessoas idosas. Diversos instrumentos podem facilitar a identificação dessa condição em pacientes sob cuidados médicos-cirúrgicos e apoiar profissionais de saúde na tomada de decisões clínicas e na adoção de protocolos específicos, funcionando como uma barreira para evitar eventos adversos e danos graves^(17)^.

A classificação das causas imediatas foi predominante no presente estudo, envolvendo profissionais da linha de frente na assistência direta ao paciente, como os de enfermagem, medicina e fisioterapia. Nesse sentido, a cultura de segurança pode refletir valores e atitudes que determinam a forma como os profissionais exercem seu trabalho e se associam ao processo de melhoria na qualidade dos serviços, incorporando estratégias e intervenções preventivas para reduzir os eventos^(18)^. Com relação às causas latentes, pesquisadores mencionam condições de trabalho, como estrutura e processos, que expõem os profissionais de saúde aos potenciais riscos, em geral evitáveis, exigindo, portanto, atenção dos dirigentes para implementação de uma gestão de risco ativa e eficaz^(19)^.

Nesse sentido, infere-se a necessidade de incluir recursos de barreiras e gatilhos relacionados ao perfil dos pacientes e identificar a natureza do dano relacionado aos eventos adversos comumente encontrados. Destaca-se, a partir dos achados, que a não identificação da deterioração clínica e o atraso na realização da intervenção cirúrgica culminaram em morte inesperada do paciente. Revisão sistemática com metanálise investigou a relação entre eventos adversos, características dos pacientes e características dos hospitais, identificando uma incidência de 8,6 eventos a cada 100 internações, dos quais 50% eram preveníveis. Entre pacientes idosos, destacaram-se os associados ao procedimento cirúrgico, ao uso de medicamentos e às infecções relacionadas à assistência^(20)^, aspectos analisados na presente pesquisa.

Para a aprendizagem organizacional, o reconhecimento, por parte dos profissionais, do que constitui um evento adverso e a segurança para realizar a notificação, sem medo de punições, são condições basilares, visto que a maioria das notificações foi realizada por algum profissional de saúde. Atualmente, há maior conscientização sobre incidentes e eventos adversos, com lideranças focadas na qualidade e segurança do paciente. Esclarecer as causas e implementar barreiras para prevenir recorrências com o envolvimento da equipe assistencial é essencial^(21)^.

A gestão do incidente resulta em consequências negativas para todos envolvidos e para a organização, além de aumento de custos, uso de recursos estruturais e materiais e maior permanência do paciente na instituição. Estudo realizado no Japão reforça que os relatórios sobre segurança do paciente objetivam construir melhores políticas que promovam prosperidade, igualdade, oportunidade e bem-estar. Esse mesmo estudo destacou a importância de investigar a relação custo-eficácia de medidas de segurança, visto que 15% dos gastos de um hospital são atribuídos ao tratamento de falhas relacionadas à assistência, como lesões intraoperatórias, administração incorreta de medicamentos e quedas^(1)^. Assim, ressalta-se a importância de abordar a segurança do paciente não apenas como um problema de saúde pública, mas também sob a ótica econômica e da sustentabilidade financeira.

Outro aspecto fundamental é o papel do paciente e da família como agentes do próprio cuidado, sobretudo no protagonismo e no reconhecimento dos riscos presentes na execução do cuidado pelos profissionais. Quando se adota um modelo de cuidado cuja centralidade é o paciente e família, reconhece-se sua participação na garantia da qualidade assistencial^(22,23)^.

Como análise nuclear desta pesquisa, salienta-se que as ações de melhoria relacionadas aos profissionais e para reduzir os riscos dos fatores organizacionais são incipientes, visto que grande parte da descrição do evento e de sua análise não elencou quais ações foram implementadas após o incidente. Ainda que o processo de acreditação traga elementos que promovam a cultura de segurança, como o registro e a análise das ocorrências, as ações para sua mitigação devem ser mais contundentes com barreiras robustas e monitoradas após o evento^(15,19)^.

Destaca-se também o uso amplo do conceito de cultura de segurança a partir da CISP, o que dificulta a definição clara do que mede a cultura de segurança e de sua presença ou ausência. Embora a instituição e seus profissionais tenham sido considerados para análise, eles assumiram a responsabilidade de identificar, comunicar e resolver questões de segurança, como os eventos adversos ocorridos^(10)^. Contudo, destaca-se uma lacuna, especialmente no que se refere à resolução do risco, que deveria envolver ações de melhoria para mitigar sua reincidência.

Ainda, houve associação entre formação e treinamento nas ocorrências de causa imediata comparada às causas latentes. Reforça-se que os profissionais aprendem a partir de suas próprias experiências. Atividades educacionais e implementação de protocolos específicos são ações de melhoria que permitem atuar como prevenção e barreira efetiva na segurança do paciente^(23)^. Reitera-se a adoção permanente de ações educativas como principal pilar na prevenção de eventos adversos, uma vez que o envolvimento direto dos profissionais de saúde no cuidado e sua mediação do processo de trabalho, a partir da formação contínua, proporciona segurança para pacientes e para eles mesmos. Profissionais de saúde, apesar de encontrarem dificuldade no processo de notificação relacionada ao sistema ou fatores inerentes à cultura organizacional, reconhecem que seu registro subsidia a educação permanente^(5)^.

Ressalta-se que, embora um programa robusto de treinamento seja oferecido aos profissionais, a percepção individual e a retenção de conhecimento variam, pois cada um traz consigo diferentes perspectivas e práticas arraigadas, de acordo com sua categoria profissional. Para que trabalhem de forma harmoniosa, respeitando os limites de suas atribuições, é essencial que haja supervisão e liderança eficazes na equipe^(24)^, garantindo as condições adequadas para o cuidado.

Ao processar a análise das notificações construídas a partir de campos textuais abertos, verificou-se que o teor das notificações mencionava, além do evento em si, situações relacionadas à própria dinâmica organizacional, como estrutura de atendimento insuficiente, condições do ambiente de trabalho consideradas inadequadas, ausência de etapas de verificação relacionadas ao atendimento do paciente, erros na documentação clínica, fragilidades relacionadas ao trabalho em equipe, falhas relacionadas à comunicação com o paciente ou ao sistema de informação e ausência de avaliação de risco, entre outras situações que poderiam constituir barreiras para a prevenção do evento. Embora tais causas possam ser consideradas genéricas, contribuem para a ocorrência de incidentes por fazerem parte do cotidiano da instituição, colocando em risco o cuidado ao paciente e envolvendo os profissionais da linha de frente^(25)^.

Diante dos dados ausentes nas análises para a composição das variáveis deste estudo, reforça-se a importância de se ter um sistema informatizado com relatórios estruturados, diretrizes claras sobre o que e como relatar, quais ações foram implementadas e seu monitoramento, com indicadores específicos para o desenvolvimento organizacional, cujos dados possam gerar aprendizagem significativa. É preciso também que o sistema seja fácil de usar e forneça *feedback* para a comunidade em geral^(3,7)^. Ainda, a demora na devolutiva após análise da notificação pode retardar medidas preventivas^(7)^.

Desse modo, o sistema de notificação deve ser construído para facilitar o relato daquele que o faz e direcionado para análise posterior, caso se almeje a aprendizagem organizacional^(6,26)^. Sugerem-se uma estrutura híbrida, com listas suspensas para dar objetividade ao relato, e os campos abertos, para que sejam utilizados para complementar as informações. Nesta pesquisa, identificou-se que o relato em um sistema com apenas campos abertos pode negligenciar informações importantes que não são registradas. Estratégias para aumentar as notificações e sua compreensão são encontradas na literatura, a título de exemplo, um hospital conseguiu incentivar a notificação dos eventos adversos e estabelecer novo canal de comunicação para melhoria do ambiente organizacional por meio de *QR Code* ^(27)^.

Apesar da importância das notificações por sistema, estudo destaca a importância de se obter informações sobre eventos adversos também por meio de outras metodologias, como o *Global Trigger Tool* ou análise retrospectiva de prontuário^(28)^.

### Limitações do estudo

O estudo em questão alcançou seu objetivo de analisar as notificações de incidentes classificados como graves e óbitos no sistema de informações sobre segurança do paciente, bem como compreender a aprendizagem institucional. Entre suas limitações, estão a análise de eventos notificados de forma narrativa, em campo aberto, que permite inúmeros desvios e distanciamentos do fato objetivo, bem como a incorporação de julgamentos de valor pelos profissionais que as fazem, nem sempre elucidando o ocorrido e as condutas tomadas. Ainda, apesar da preocupação em uniformizar a classificação e solucionar divergências entre os pesquisadores, é impossível anular todos os vieses pela natureza da análise, que é ancorada em valores pessoais e experiências prévias.

### Contribuições para a Área

Ao analisar as notificações de eventos adversos que resultaram em dano grave ou óbito, é possível identificar pontos críticos e vulnerabilidades nos processos assistenciais, administrativos ou de gestão. Contudo, essa identificação nem sempre se traduz em oportunidades de aprendizagem e em ações de melhoria após o evento, o que fortalece a cultura de segurança.

## CONCLUSÕES

As notificações de incidentes analisadas, com desfechos graves e óbitos de sistema de informações próprio de organização acreditadora, evidenciaram que, embora não haja modelo único desses sistemas, a ausência de critérios para documentar o incidente impacta a análise pós-evento e dificulta compreender como ocorreu a aprendizagem organizacional.

Na ocorrência de eventos adversos, há uso adicional dos recursos. As ações de melhoria estavam ausentes na maioria das análises, identificando fragilidades nos processos de aprendizagem e na maturidade organizacional. Em geral, as ações de melhoria relacionadas aos profissionais se caracterizaram como orientações informais, e as ações de melhoria relacionadas à organização são referentes à cultura de segurança, embora isso pode significar uma diversidade de ações.

A classificação do tipo de causa como imediata foi predominante, e ela envolveu os profissionais da linha de frente na assistência direta ao paciente, não identificando-se descrição de ações de apoio aos profissionais em relação ao desfecho e ao sofrimento decorrente.

Embora haja vantagens de o sistema de notificação permitir que as notificações sejam realizadas de forma narrativa, para explicar com riqueza de detalhes o que esteve envolvido na sua ocorrência, esse formato apresenta como desvantagem a dificuldade em sua análise, para identificação das ações necessárias para evitar a sua recorrência. Dessa forma, reforça-se a importância de que o sistema de notificação seja estruturado de maneira a facilitar tanto o relato quanto a análise posterior das informações, promovendo a melhoria contínua dos processos e o aprendizado organizacional.

## Data Availability

Os dados de pesquisa não estão disponíveis.
